# Role of Non-Coding RNAs in Post-Transcriptional Regulation of Lung Diseases

**DOI:** 10.3389/fgene.2021.767348

**Published:** 2021-11-08

**Authors:** Dharmendra Kumar Soni, Roopa Biswas

**Affiliations:** Department of Anatomy, Physiology and Genetics, School of Medicine, Uniformed Services University of the Health Sciences, Bethesda, MD, United States

**Keywords:** microRNA, long noncoding RNA, asthma, chronic obstructive pulmonary disease, cystic fibrosis, idiopathic pulmonary fibrosis

## Abstract

Non-coding RNAs (ncRNAs), notably microRNAs (miRNAs) and long noncoding RNAs (lncRNAs), have recently gained increasing consideration because of their versatile role as key regulators of gene expression. They adopt diverse mechanisms to regulate transcription and translation, and thereby, the function of the protein, which is associated with several major biological processes. For example, proliferation, differentiation, apoptosis, and metabolic pathways demand fine-tuning for the precise development of a specific tissue or organ. The deregulation of ncRNA expression is concomitant with multiple diseases, including lung diseases. This review highlights recent advances in the post-transcriptional regulation of miRNAs and lncRNAs in lung diseases such as asthma, chronic obstructive pulmonary disease, cystic fibrosis, and idiopathic pulmonary fibrosis. Further, we also discuss the emerging role of ncRNAs as biomarkers as well as therapeutic targets for lung diseases. However, more investigations are required to explore miRNAs and lncRNAs interaction, and their function in the regulation of mRNA expression. Understanding these mechanisms might lead to early diagnosis and the development of novel therapeutics for lung diseases.

## Introduction

Non-coding RNAs (ncRNAs) are non-protein-coding RNA transcripts and were initially believed as “non-functional parts” and/or “junk RNAs” and/or “dark matter” of the human genome. But, the discoveries of the transcribed regions and protein-coding genes, i.e., approximately 85 and 2%, respectively, reveal that only a small portion of the human transcriptome encode for protein and the majority are non-protein-coding (Lander et al., 2001; Djebali et al., 2012; Hangauer et al., 2013; Jensen et al., 2013). This assessment has subverted the aforementioned conception and highlights the significance of ncRNAs, which leads to a paradigm shift and scientific revolution in RNA biology and regulation. Today, there is enormous evidence proving the function of ncRNAs as versatile key regulators of epigenetics, transcription, post-transcription, and translation (Cech and Steitz, 2014; Peschansky and Wahlestedt, 2014; Zhang et al., 2019). The pivotal role of ncRNAs in the regulation of nearly all biological activities, from tissue repair to organ development and immunity, is well-established. Consequently, deregulation in ncRNA networks has been associated with a broad spectrum of pathological conditions and human diseases including lung diseases (Taft et al., 2010; Esteller, 2011; Beermann et al., 2016; Groot et al., 2018; Bao et al., 2019; Bhatti et al., 2021).

Lung diseases are a leading public health concern and cause substantial morbidity and mortality, globally (Schluger and Koppaka, 2014; Glass and Rosenthal, 2018). Undoubtedly, this necessitates in-depth knowledge of the lung disease etiology and pathophysiology, with a focus on inventing more efficacious therapeutic approaches. In recent decades, several reports have established the association of ncRNAs in various lung diseases and their pivotal functions in lung development and homeostasis (Lu et al., 2018; Pattarayan et al., 2018; Wang et al., 2019), expediting a new paradigm for lung disease diagnosis, control, and treatment. Here, we provide a comprehensive overview of the post-transcriptional regulation of ncRNAs, with special emphasis on microRNAs (miRNAs) and long ncRNAs (lncRNAs), in lung diseases such as asthma, chronic obstructive pulmonary disease (COPD), cystic fibrosis (CF), and idiopathic pulmonary fibrosis (IPF). Alterations of miRNA and lncRNA expression level in the disease state compared to the normal state could be exploited to identify biomarkers and targets for drug development. Understanding how post-transcriptional mechanisms regulate lung diseases will lead to the development of candidate therapeutic targets for the early diagnosis and treatment of lung diseases.

### MicroRNAs and Long ncRNAs

The ncRNA repertoire encompasses myriads of RNA species and according to their regulatory roles are broadly classified into two categories, housekeeping and regulatory ncRNAs. The regulatory ncRNAs further consist of diverse groups of ncRNAs with the two-utmost noteworthy, microRNAs (miRNAs, transcripts between 19 and 25 nucleotides) and long ncRNAs (lncRNAs, transcripts >200 nucleotides). MiRNAs generally negatively regulate gene expression in a sequence-specific way at the post-transcriptional stage either through the target messenger RNA (mRNA) cleavage and degradation, and/or by inhibition of translation. On the other hand, lncRNAs are divided into different types and regulate either negatively or positively each stage of gene expression *via* the interactions with DNA, RNA, or protein and through various mechanisms (Fatica and Bozzoni, 2014; Chew et al., 2018). The biogenesis, characteristics, types, and mechanism of action of miRNAs and lncRNAs have been described in multiple articles (Denli et al., 2004; Han et al., 2004; Du and Zamore, 2005; Kim, 2005; Bartel, 2009; Kugel and Goodrich, 2012; Fatica and Bozzoni, 2014; Quinn and Chang, 2016; Kopp and Mendell, 2018; Zhang et al., 2019; Statello et al., 2021). Collectively, both of these ncRNA species have an imperative role in development and homeostasis as well as in diseases.

### Non-Coding RNAs in Lung Disease

Emerging evidence suggests that in the respiratory system, ncRNAs are accountable for normal lung development and maintenance of lung homeostasis. Thus, deregulation of miRNAs and lncRNAs causes pathophysiological alteration of the respiratory system leading to the initiation, progression, and development of various types of lung diseases. In the following sections, we have described the emerging roles and mechanistic functions of miRNA and lncRNA in various lung diseases such as asthma, chronic obstructive pulmonary disease (COPD), cystic fibrosis (CF), and idiopathic pulmonary fibrosis (IPF).

### Asthma

Asthma is a multifaceted heterogeneous disease, primarily characterized by chronic inflammation, hyperresponsiveness, and transient airflow obstruction of the airways. The global increase in the incidence of asthma has been reported in all age groups, and approximately 300 million people are affected by asthma (Cevhertas et al., 2020; Stern et al., 2020). Therefore, the management of asthma as well as developing novel therapies is vital.

Several ncRNAs regulate airway inflammation and are associated with the pathophysiology of asthma. For example, upregulation of miR-221 and miR-485-5p are reported in the blood sample of asthmatic children (Liu et al., 2012). In a murine model of asthma, miR-221 and miR-485-5p regulate interleukin-5 (IL-5) by targeting sprouty-related protein with an EVH1 domain-2 (*Spred-2*), which negatively regulates the Ras/ERK pathway involved in a variety of cellular processes, including airway inflammation and hypersensitivity (Liu et al., 2012). Elevated level of miR-1248 has been reported in the serum of asthmatic patients and it induces increased expression of IL-5 and upregulation of Th2 cytokine through the direct interaction with *IL-5* (Panganiban et al., 2012). The imbalance of Th1/Th2 cytokines has been found as a predominant factor associated with asthma, where increased expression of Th2 cytokines, mainly IL-4, IL-5, IL-9, and IL-13 promote the serum immunoglobulin E (IgE) and eosinophilia that stimulate a variety of cellular processes, including mucus hypersecretion, airway inflammation, and hypersensitivity (Ngoc et al., 2005; Zhu et al., 2016). On the other hand, Th1 cytokines, such as IFN-γ and IL-12, play an antagonist role in IgE synthesis as well as other Th2 responses. Thus, restoration of Th1/Th2 balance *via* inhibition of Th2 cytokines and activation of Th1 cytokines is one of the critical aspects in the treatment of asthma. One of the most studied miRNAs in asthma is miR-21. Several studies with asthmatic mice model and in asthmatic children indicate the upregulation of miR-21 negatively regulates IL-12p35, signal transducer and activator of transcription 4 (STAT4), phosphatase and tensin homolog deleted on chromosome 10 (PTEN), and histone deacetylase 2 (HDAC2), and positively regulates phosphoinositide 3-kinase (PI3K), which may promote increased expression of Th2 cytokines and inhibit Th1 cytokines expression (Lu et al., 2009; Wu S.-Q. et al., 2014; Wu XB. et al., 2014; Liu et al., 2015; Perry et al., 2015; Sawant et al., 2015; Elbehidy et al., 2016; Kim et al., 2017; Hammad Mahmoud Hammad et al., 2018). Thereby, the role of miR-21 in controlling the Th1/Th2 ratio, airway hypersensitivity, and cell proliferation and migration is established in different asthmatic models. In the bronchial epithelium of neutrophilic asthmatic, miR-629-3p is upregulated and induces neutrophil chemoattractant IL-8, which suggests its role in the airway neutrophilia and disease pathogenesis through the regulation of proinflammatory and wound-repair pathways (Maes et al., 2016). In lung tissues from allergic asthma patients and ovalbumin (OVA)-induced mice, upregulation of miR-943-3p and downregulation of its target secreted frizzled-related protein 4 (*SFRP4*) enhances airway inflammation progression and remodeling *via* the activation of Wingless/Integrase I (Wnt) signaling pathway (Shen et al., 2019). The importance of WNT signaling has been shown in the development of the organism, context-dependent transcription of targets genes, maintaining equilibrium among proliferation and differentiation of airway smooth muscle (ASM) progenitor cells, and asthma pathogenesis (Sharma et al., 2010; Choy et al., 2011; Wang et al., 2013; Carraro et al., 2014; Barreto-Luis et al., 2017).

The decreased expression of miR-181b-5p is reported in plasma and epithelium of eosinophilic asthmatic and it has been demonstrated that miR-181b-5p negatively regulates proinflammatory cytokines, IL-1β and C-C motif chemokine ligand (CCL)-11 (eotaxin-1) expression by binding to its target secreted phosphoprotein-1 (SPP-1), which is associated with the recruitment of eosinophils into airways (Huo et al., 2016). In the lungs of asthmatic mice, miR-20b promotes the elevation of CCL-2 concentration and accumulation of myeloid-derived suppressor cells, which suppresses the Th2 response and airway inflammation in a transforming growth factor-beta (TGF-β)-dependent manner (Ma et al., 2017a; Ma et al., 2017b). MiR-221-3p is downregulated in the airway epithelium and sputum of asthmatics and its downregulation suppresses inflammatory cytokine, chemokine CCL-24 (eotaxin-2) and CCL-26 (eotaxin-3) expression, which are involved in the migration of eosinophils into the airways, by inducing the expression of its target chemokine C-X-C motif ligand (CXCL)-17, an anti-inflammatory chemokine (Zhang K. et al., 2018). This suggests the protective role of miR-221-3p against airway eosinophilic inflammation. The reduced expression of miR-485 is observed in the mouse model of chronic asthma. Consistently, overexpression of miR-485 leads to reduced proliferation of airway smooth muscle cells (ASMCs) and induces apoptosis by targeting Smad ubiquitin regulatory factor 2 (*Smurf2*) (Wang et al., 2018). Smurf2 modulates the TGF-β/decapentaplegic homolog (Smads) signaling pathway, which is shown to be associated with the remodeling of the airway in asthma (Qu et al., 2012). The association of miR-142-3p with WNT signaling and maintaining equilibrium among proliferation and migration of ASMCs has been observed in bronchial biopsies of asthmatics (Bartel et al., 2018). In bronchial epithelial cells from asthmatic patients, decreased level of miR-744 induces cell proliferation by targeting *TGF-ß1* and regulating the Smad3 pathway (Huang et al., 2019). The downregulation of miR-30a and upregulation of its target autophagy-related 5 (*ATG5*) is reported in lung tissues from asthmatic children and in mice treated with OVA, and promotes fibrogenesis, autophagic flux, and airway remodeling (Li et al., 2020).

In addition to miRNAs, lncRNAs are also associated with the regulation of airway inflammation and asthma. For example, in the rat model of asthma, upregulation of brain cytoplasmic RNA 1 (BCYRN1) lncRNA targets canonical transient receptor potential 1 (*TRPC1*), which is implicated in the pro-proliferative and pro-migratory role of BCYRN1 and induces proliferation and migration of ASMCs (Zhang XY. et al., 2016). TRPC1 has been reported as an important molecular counterpart of Ca2+ channels in ASMCs and as a critical component contraction and proliferation of vascular smooth muscle cells (Ong et al., 2003; Dietrich et al., 2006). A similar study, using rat model asthma, demonstrated that miR-150 downregulates BCYRN1 and reduces proliferation and migration of ASMCs (Zhang X.-y. et al., 2017). PVT1 lncRNA is upregulated in severe asthmatic patients who are insensitive to corticosteroids, and induces IL-6 expression and proliferation of ASMCs (Austin et al., 2017). The upregulation of TCF7 lncRNA and TIMMDC1 and the role of TCF7 in the regulation of TIMMDC1 expression and proliferation and migration of ASMCs have been established in asthmatics (Fan et al., 2019). Similarly, TUG1 and MALAT1 lncRNAs induces proliferation and migration of ASMCs *via* targeting miR-590-5p (TUG1/miR-590-5p/FGF1 axis) and miR-150 (miR-150-eIF4E/Akt signaling), respectively (Lin J. et al., 2019; Lin L. et al., 2019). The upregulation of the lncRNA antisense non-coding RNA in the INK4 locus (ANRIL)/miR-125a axis is found especially in the plasma of bronchial asthmatics at exacerbation (BA-E) compared to bronchial asthmatics at remission (BA-R) and control groups (Ye et al., 2020). Furthermore, there is a positive correlation between this regulatory axis and pro-inflammatory cytokines (TNF-α, IL-1β, IL-6, and IL-17) in bronchial asthmatics. A brief summary of ncRNAs, miRNAs and lncRNAs, associated with asthma are shown in Table 1. From these studies, it is clear that through the various mechanisms including post-transcriptional regulation miRNAs, lncRNAs and associated molecules play a pivotal role in the genesis and development of asthma. Further analyses of the function, and mechanism of action of ncRNAs will lead to therapeutic targets for asthma.

**TABLE 1:** List of ncRNAs and their targets and functions in asthma.

ncRNA	Source	Expression	Target/regulator	Function	Reference
miR-21	doxycycline-induced lung-specific IL-13 transgenic mice	up	IL-12p35	modulates IL-12 and Th cell polarization	Lu et al. (2009)
miR-1248	serum from asthmatics	up	IL-5	positive regulator to increase IL-5	Panganiban et al. (2012)
miR-21	bronchial epidermal cells from asthmatic treated with or without inhaled corticosteroids (ICS)	up	IL-13	positively correlates with IL-13	Wu et al. (2014b)
miR-126					
miR-21	lung tissue from allergic asthmatic mice model	up	IL-12, STAT4	develops allergic asthma	Wu et al. (2014a)
miR-21	HASM cells	up	PTEN	triggers cell proliferation and migration	Liu et al. (2015)
miR-155	HASM cells from asthmatic	up	COX-2	positively correlates with COX-2	Comer et al. (2015)
miR-21	serum from asthmatic children without ICS, steroid sensitive (SS) asthma children and steroid resistant (SR) asthma children	up	IL-12p35	negatively correlates with serum IL-12p35 and FEV1, while positively correlates with both sputum and blood eosinophils	Elbehidy et al. (2016)
miR-146a	plasma from asthmatic children	up	EGFR	inhibits proliferation and promotes apoptosis of BSMCs	Zhang et al. (2016b)
miR-181b-5p	epithelial cells and plasma from asthmatic	down	SPP1	regulates IL-13-induced IL-1β and CCL11	Huo et al. (2016)
miR-21	lung cells from severe, steroid-insensitive allergic asthmatic mice model	up	phosphatase and tensin homolog	Ant-21 treatment reduces PI3K activity and restores HDAC2 as well as suppresses airway hyperresponsiveness and restores steroid sensitivity	Kim et al. (2017)
miR-155	lung tissue from allergic asthmatic mice model	up	IL-33	regulates ILC2s and IL-33	Johansson et al. (2017)
miR-371	CD4^+^ T cells from asthmatic	up	Runx3	regulates Runx3 in a combinatorial manner and modulates Th1/Th2 balance	Qiu et al. (2017)
miR-138					
miR-544					
miR-145					
miR-214					
miR-98	peripheral B cells from allergic asthmatic	up	TSP1, IL-13	suppresses TSP1	Chen et al. (2017)
miR-21	plasma from asthmatic children with ICS	up	IL-13	miR-21 positively correlates with IL-13 and eosinophil percentage, while miR-146a only correlates to eosinophil percentage	Hammad Mahmoud Hammad et al. (2018)
miR-146a					
miR-221-3p	bronchial brushings, induced sputum, and plasma from steroid-naive asthmatic	down	CXCL17	regulates CCL24, CCL26, and airway eosinophilic inflammation	Zhang et al. (2018b)
miR-485	ASMCs from mouse model of chronic asthmatic	down	Smurf2	regulates cell proliferation and apoptosis	Wang et al. (2018)
miR-192	plasma and CD4^+^ T cells from acute asthmatic children	down	CXCR5	blocks T follicular helper cells activation pathway	Zhang et al. (2018a)
miR-943-3p	lung tissues from allergic asthmatics and OVA-induced mice	up	SFRP4	enhances airway inflammation progression and remodeling	Shen et al. (2019)
miR-744	bronchial epithelial cells from asthmatic	down	TGF-ß1	induces cell proliferation through mediating Smad3 pathway	Huang et al. (2019)
miR-30a	lung tissues from asthmatic children and OVA-induced mice	down	ATG5	induces fibrogenesis, autophagic flux and airway remodeling	Li et al. (2020)
lncR-BCYRN1	ASMCs from rat asthmatic model	up	TRPC1	induces cell proliferation and migration	Zhang et al. (2016a)
lncR-BCYRN1	ASMCs from rat asthmatic model	up	miR-150	regulates cell proliferation and migration	Zhang et al. (2017c)
lncR-PVT1	ASMCs from severe asthmatics	up	IL-6	regulates IL-6 and cell proliferation	Austin et al. (2017)
lncR-TCF7	ASMCs from asthmatics	up	TIMMDC1	regulates cell growth and migration	Fan et al. (2019)
lncR-MEG3	peripheral blood CD4 + T cells from asthmatics	up	miR-17	regulates RORγt and affects Treg/Th17 balance	Qiu et al. (2019)
lncR-TUG1	ASMCs from rat asthmatic model	up	miR-590-5p	regulates cell proliferation and migration	Lin et al. (2019a)
lncR-MALAT1	ASMCs from asthmatics	up	miR-150	derepresses eIF4E, activates Akt signaling, and regulates cell proliferation and migration	Lin et al. (2019b)
lncR-ANRIL	plasma from bronchial asthmatics	up	miR-125a	positive correlations with pro-inflammatory cytokines (TNF-α, IL-1β, IL-6, and IL-17)	Ye et al. (2020)

### Chronic Obstructive Pulmonary Disease (COPD)

COPD is a heterogenous persistent lung disease, caused by progressive and irreversible airflow obstruction. COPD has a high rate of morbidity and mortality, accounting for 3.2 million deaths globally, and is considered the third leading cause of death (WHO, 2021). Among the environmental factors, the recurrent exposure of noxious particles and gas irritants such as cigarette smoke to the lungs are among the main causes of the development of COPD. However, genetic and epigenetic factors also play an important role in the pathogenesis of COPD, as this disease is reported in only 20% of smokers.

The involvement of ncRNAs in the pathogenesis and development of COPD is established by several studies. For example, upregulation of miR-15b and downregulation of its target *SMAD7*, which is an inhibitory SMAD in TGF-β signaling, is reported in lung tissues of COPD patients compared with smokers without obstruction, and thereby regulates TGF-β signaling pathway and pathogenesis of COPD (Ezzie et al., 2012). TGF-β is a profibrogenic cytokine and the impairment in TGF-β signaling in COPD patients induces fibrotic airway remodeling that could promote a decline in lung function (Morty et al., 2009). MiR-135b is upregulated in lung tissues of mice exposed to cigarette smoke, and regulates the IL-1 pathway by targeting IL-1R1 (Halappanavar et al., 2013). Several studies have shown the involvement of IL-1 signaling in chronic inflammation, remodeling of airways, and pathogenesis of COPD (Osei et al., 2020). The upregulation of miR-223 in lung tissues of COPD patients and in mice exposed to cigarette smoke is inversely correlated to the expression of its target *HDAC2* and leads to the upregulation of CX3CL1 (Leuenberger et al., 2016). Declined HDAC activity permits the acetylated chromatin to be unbound to histones and this step allows chromatin access for transcription factors and transcription of various inflammatory cytokines and chemokines (Barnes et al., 2005). Elevated expression of miR-195 is observed in lung tissues of COPD patients and mice exposed to cigarette smoke, which causes downregulation of its target PH domain and leucine-rich repeat protein phosphatase 2 (*PHLPP2*) and increases Akt phosphorylation, leading to increased expression of IL-6 and TNF-α (Gu et al., 2018). Earlier studies suggest the role of PHLPP2 in direct dephosphorylation and inactivation of Akt, which has multifunctional activities and is a potential regulator of various cellular processes involved in the pathogenesis of COPD (Bozinovski et al., 2006; Gao et al., 2009; Nowak et al., 2015). The upregulation of miR-664a-3p and downregulation of its target four and a half LIM domains 1 (*FHL1*), which acts as a transcription factor and implicated in various cellular mechanisms, in lung tissue and peripheral blood mononuclear cells (PBMCs) of COPD patients positively correlated with forced expiratory volume in one second (FEV1)/forced vital capacity (FVC)% and has a role in cigarette smoke-induced COPD (Zhong et al., 2019). A recent report demonstrates that the upregulation of miR-130 in BEAS-2B cells treated with cigarette smoke extract (CSE) and in mice exposed to CSE, negatively regulates Wnt/β-catenin signaling by targeting Wnt1 and modulating β-Catenin, and lymphoid enhancer-binding factor (LEF) (Wu et al., 2020). Earlier, the role of β-Catenin is shown in cell proliferation and injury repair (Zemans et al., 2011; Tanjore et al., 2013). Further, it has been demonstrated that activation of Wnt/β-catenin signaling may potentially attenuate COPD pathogenesis (Kneidinger et al., 2011; Uhl et al., 2015).

Reduced expression of miR-34c in lung tissues of COPD patients modulates the expression of SERPINE1, which is a protease and fibrinolysis inhibitor (Savarimuthu Francis et al., 2014). The authors suggest that SERPINE1 has other functions apart from antiproteases in the lung that may play important role in emphysema progression. Nuclear factor-kappaB (NF-κB) is a crucial transcription factor and persistent stimulation of the NF-κB signaling pathway provokes the exaggerated synthesis of pro-inflammatory mediators such as IL-8 and TNF-α, which leads to airway impairment in COPD patients (Edwards et al., 2009). The downregulation of miR-149-3p in the blood of smokers with COPD activates TLR-4/NF-κB signaling and upregulates IL-1β and TNF-α by targeting *TLR-4* (Shen et al., 2017). Moreover, miR-145-5p expression is reduced in lung tissues of smokers without or with COPD and regulates p53-mediated apoptotic signaling, NF-κB signaling, TNF-α, IL-6, and IL-8 by targeting kruppel like factor 5 (*KLF5*) (Dang et al., 2019). Consistently, overexpression of miR-145-5p attenuates CSE-stimulated apoptosis and inflammation in human bronchial epithelial cells (HBECs) (Dang et al., 2019). The role of p53-mediated signaling pathways has been shown in CSE-induced cell apoptosis (Lee and Choi, 2018). KLF5 belongs to a family of zinc-finger (ZF) containing transcription factors and is implicated in the regulation of a wide range of cellular processes such as cell proliferation, apoptosis, inflammation, migration, and differentiation (Dong and Chen, 2009). The downregulation of miR-29b is found in lung tissues and plasma from COPD patients, which regulates CSE-induced IL-8 expression by targeting bromodomain protein 4 (*BRD4*) (Tang et al., 2019). The role of BRD4 has been shown in direct or indirect regulation of gene transcription (Devaiah et al., 2016a; Devaiah et al., 2016b). Further, studies also demonstrated that inhibition of BRD4 significantly decreases the level of proinflammatory cytokines, which suggests its important role in the inflammatory process (Nicodeme et al., 2010; Tian et al., 2017). Thus, signify the vital function of the miR-29b-BRD4 axis in airway inflammation and pathogenesis of COPD.

In addition to miRNAs, the association of lncRNA is also shown in the pathogenesis and development of COPD. For example, in the lung tissues of COPD patients, TUG1 is upregulated and its silencing reduces α-SMA and fibronectin expression and stimulates the proliferation of TGF-β induced- BEAS-2B and HFL1 cells (Tang W. et al., 2016). The upregulation of lncRNA-ENST00000502883.1 is found in B cells and CD4^+^ T cells from COPD patients and it is shown that it affects PBMC recruitment *via* regulation of CXCL16 (Qu et al., 2018). CXCL16 functions as a chemoattractant for Th1 cells and it is considered as a systemic inflammatory marker for COPD (Shashkin et al., 2003; Donnelly and Barnes, 2006; Eagan et al., 2010). The nuclear enriched abundant transcript 1 (NEAT1) is upregulated in plasma from COPD patients and negatively correlates with miR-193a and positively correlates with GOLD stage and the expressions of TNF-α, IL-1β, IL-6, and IL-17 (Ming et al., 2019). NEAT1-induced inflammatory cascades and oxidative stress lead to severe lung injury, which establishes NEAT1 is positively correlated with COPD severity and inflammation and its potential in the prediction of disease susceptibility and acute exacerbation risk. The metastasis-associated lung adenocarcinoma transcript 1 (MALAT1) is upregulated in lung tissues of COPD patients (Hu et al., 2020). In the same study, *in vitro* experiments with TGF-β-treated human lung fibroblasts showed that MALAT1 downregulation stimulates cellular viability and inhibits mesenchymal protein expression by regulating the mTOR pathway, which is involved in lung cell senescence in COPD. The downregulation of HOXA cluster antisense RNA 2 (HOXA-AS2) is found in lung tissues from COPD patients and further studies in CSE-treated human pulmonary microvascular endothelial cells (HPMECs) demonstrated that the downregulation of HOXA-AS2 suppresses cell proliferation *via* Notch1 signaling (Zhou et al., 2020). This implies that upregulation Notch1, which is implicated in various cellular processes such as cell proliferation, differentiation, and apoptosis, stimulates HOXA-AS2-dependent cell proliferation and mitigates the cell viability injury. The lung cancer-associated transcript 1 (LUCAT1) is elevated in the serum of COPD patients (Zhao et al., 2021). Further studies in CSE-treated 16HBE cells show that LUCAT1 downregulates its target, miR-181a-5p, upregulates inflammatory cytokines (IL-1β, IL-6, and TNF-α), and regulates cell proliferation and apoptosis *via* the Wnt/β-catenin pathway (Zhao et al., 2021). The role of activated Wnt/β-catenin pathway in induction of inflammatory cytokines (IL-1β, IL-6, and TNF-α) and cell proliferation and apoptosis are well characterized (Masckauchan et al., 2005; Aumiller et al., 2013; Jang et al., 2017). This suggests LUCAT1 plays an important role in the regulation of inflammatory cytokines and the Wnt/β-catenin pathway, thus have a crucial function in the pathogenesis of COPD. Table 2 summarizes the list of ncRNAs, miRNAs and lncRNAs with their targets and functions in COPD. Collectively, these studies suggest that miRNAs, lncRNAs, and their interaction and regulation have a significant role in the pathogenesis and development of COPD. Understanding these mechanisms will lead to novel therapeutic interventions and approaches for better management of COPD.

**TABLE 2:** List of ncRNAs and their targets and functions in COPD.

ncRNA	Source	Expression	Target/regulator	Function	Reference
miR-15b	lung tissues from smokers with and without COPD	up	SMAD7	regulates TGF-β signaling	Ezzie et al. (2012)
miR-199a-5p	lung tissues from COPD patients	up	HIF-1α	regulates HIF-1α	Mizuno et al. (2012)
miR-135b	lungs tissues from mice exposed to cigarette smoke	up	IL-1R1	regulates IL-1 pathway	Halappanavar et al. (2013)
miR-34c	lung tissues from COPD patients	down	SERPINE1	regulates TGF- β signaling	Savarimuthu Francis et al. (2014)
miR-223	lung tissues from COPD patients and mice exposed to cigarette smoke	up	HDAC2	upregulates CX3CL1	Leuenberger et al. (2016)
miR-218	serum from smokers without or with COPD	down	TNFR1	upregulates MUC5AC, IL-6, IL-8, TNFR1, and p-p65	Xu et al. (2017)
miR-181c	lung tissues from COPD patients and mice exposed to cigarette smoke	down	CCN1	increases inflammatory response, neutrophil infiltration, ROS generation, and inflammatory cytokines induced by CS	Du et al. (2017)
miR-149-3p	blood from smokers without or with COPD	down	TLR-4	activates TLR-4/NF-κB signaling and upregulates IL-1β and TNF-α	Shen et al. (2017)
miR-195	lung tissues from COPD patients and mice exposed to cigarette smoke	up	PHLPP2	increases Akt phosphorylation, IL-6 and TNF-α in BEAS-2B cells	Gu et al. (2018)
miR-3202	blood from smokers without or with COPD	down	FAIM2	upregulates INF-γ, TNF-α and FAIM2 and downregulates Fas and FasL in T lymphocytes	Shen et al. (2018)
miR-664a-3p	lung tissue and PBMCs from COPD patients	up	FHL1	positively correlates with FEV1/FVC%	Zhong et al. (2019)
miR-145-5p	lung tissues from smokers without or with COPD	down	KLF5	conferred protection against CSE-induced airway epithelial cell apoptosis and inflammation	Dang et al. (2019)
miR-29b	lung tissues and plasma from COPD patients	down	BRD4	regulates CSE-induced IL-8	Tang et al. (2019)
miR-130	cigarette smoke extract (CSE)-treated BEAS-2B cells and CS-exposed mice	up	WNT1	negatively regulates Wnt/β-catenin signaling by modulating Wnt1, β-Catenin, and LEF1	Wu et al. (2020)
lncR-TUG1	lung tissues from COPD patients	up	α-SMA and fibronectins	Knockdown of lncRNA TUG1 promotes BEAS-2B and HFL1 cell proliferation after TGF-β treatment	Tang et al. (2016b)
SAL-RNA1	lung tissues from COPD patients	down	SIRT1/FoxO3a, p53, p21	regulate AECII senescence	Gu et al. (2017)
SAL-RNA2		up			
lncR-ENST00000502883.1	B cells and CD4^+^ T cells from COPD patients	up	CXCL16	effects PBMC recruitment	Qu et al. (2018)
lncR-NEAT1	plasma from COPD patients	up	miR-193a	positively correlates with GOLD stage and TNF-α, IL-1β, IL-6 and IL-17	Ming et al. (2019)
lncR-ENST00000447867	CD4^+^ T cells from acute exacerbations of COPD patients	up	RAPGEF3	affect RAPGEF3 as miRNA sponges	Qi et al. (2019)
NR-026690					
lncR-ANRIL	Plasma from acute exacerbations of COPD patients	down	TNF-α, IL-1β, IL-17A, LTB-4	associates with lower acute exacerbation risk, decreased inflammatory cytokines, and mild GOLD stage	Ge et al. (2019)
lncR-MALAT1	lung tissues from COPD patients	up	mTORC1	downregulation of MALAT1 induces cellular viability following TGF-β stimulation in HFL1 cells	Hu et al. (2020)
lncR-HOXA-AS2	lung tissues from COPD patients	down	Notch1	regulating HPMECs proliferation	Zhou et al. (2020)
lncR-LUCAT1	serums from COPD patients	up	miR-181a-5p	LUCAT1 silencing alleviates CSE’s effects on 16HBE cell proliferation and apoptosis	Zhao et al. (2021)

### Cystic Fibrosis (CF)

CF is the most common genetic autosomal recessive lethal disease. It is caused by loss-of-function mutations in the cystic fibrosis transmembrane conductance regulator (CFTR) gene leading to aberrant translation, protein mis-folding, and/or trafficking (Riordan et al., 1989; Cutting, 2015). Impairment of CFTR, a crucial chloride ion channel, results in ionic disequilibria and concurrently, airway dehydration and mucus accumulation. This further leads to chronic airway infections and inflammation and eventually, fatal deterioration in lung function.

Growing evidence supports the role of miRNAs in the direct or indirect regulation of CFTR and/or CFTR-related genes/proteins (Table 3). For example, several miRNAs including miR-101, miR-145, miR-384, miR-494, miR-600 are directly concomitant with CFTR dysregulation in airway epithelial cells like A549, Beas-2B, bronchial brushings, Caco-2, Calu-3, CFBE41o-, differentiated primary cell cultures, 16HBE14o-, HBEpiC, HEK293, PANC-1 (Gillen et al., 2011; Megiorni et al., 2011; Hassan et al., 2012; Oglesby et al., 2013; Ramachandran et al., 2013; Viart et al., 2015; Fabbri et al., 2017; Lutful Kabir et al., 2018). These studies suggest that the regulation of CFTR expression by miRNAs in different cell types is diverse, tissue-specific, and time-dependent. Antisense targeting of miR-145-5p through peptide nucleic acid (PNA) upregulates CFTR expression (Fabbri et al., 2017; Finotti et al., 2019). Consistently, suppression of miR-145 has been shown to restore F508del CFTR expression (Lutful Kabir et al., 2018; Dutta et al., 2019). Further, a recent study shows PNA masking of the miR-145-5p binding site of CFTR mRNA upregulates CFTR at both mRNA and protein levels (Sultan et al., 2020). Similar PNA targeting of miR-101-3p also upregulates CFTR (Fabbri et al., 2021). The indirect association is also determined between miRNA and CFTR. For example, miRNA-138 interacts with its target switch-independent 3 homolog A (*SIN3A*), a transcriptional regulatory protein, and downregulates CFTR (Ramachandran et al., 2012). Further, the same study showed that controlling miR-138/SIN3A expression restores F508del-CFTR expression. The upregulation of miR-9 in CF cells downregulates its target anoctamin 1 (*ANO1*) alias calcium-activated chloride channel (transmembrane protein 16A, TMEM16A) and preventing the inhibition of ANO1 *in vitro* and *in vivo* CF models *via* the miR-9 target site blocker (TSB) elevates chloride efflux, mucociliary clearance, and migration rate of cells (Benedetto et al., 2017; Sonneville et al., 2017).

**TABLE 3:** List of ncRNAs that directly or indirectly target and regulate CFTR in cystic fibrosis.

ncRNA	Source	Target/regulator	Function	Reference
miR-101, miR-145, miR-384, miR-494, miR-600	A549, Beas-2B, bronchial brushing, Caco-2, Calu-3, CFBE41o-, differentiated primary cell cultures, 16HBE14o-, HBEpiC, HEK293, PANC-1	CFTR	directly target and regulate CFTR	Gillen et al. (2011); Megiorni et al. (2011); Hassan et al. (2012); Oglesby et al. (2013); Ramachandran et al. (2013); Viart et al. (2015); Fabbri et al. (2017); Lutful Kabir et al. (2018); Dutta et al. (2019); Finotti et al. (2019); Sultan et al. (2020); Fabbri et al. (2021)
miRNA-138	differentiated primary cell cultures, Calu-3, HEK293, HeLa	SIN3A	regulates CFTR	Ramachandran et al. (2012)
miR-9	CFBE41o, 16HBE14o-	ANO1/TMEM16A	modulates mucus hydration and chloride efflux activity	Sonneville et al. (2017)
lncR-BGas	CFPAC, 1HAEo-, 16HBE14o-, CFBE41o-	CFTR	directly targets and regulates CFTR	Saayman et al. (2016)

MiRNAs that regulate CF lung disease *via* the regulation of inflammation, airway obstruction, or infection are listed in Table 4. For example, the elevated expression of miR-155 in the CF lung epithelium leads to downregulation of SH-2 containing inositol 5′ polyphosphatase 1 (SHIP1), an inositol 5-phosphatase, and thereby induces IL-8 expression *via* regulation of phosphatidylinositol-3 kinase/protein kinase B (PI3K/Akt) signaling (Bhattacharyya et al., 2011). Further, the RNA-binding protein tristetraprolin (TTP), a zinc finger protein also known as ZFP36, suppresses miR-155 expression in CF lung epithelial cells *via* upregulation of miR-1, while KH-type splicing regulatory protein (KSRP), the KH domain-containing splicing factor, upregulates miR-155 *via* promoting enhanced biogenesis (Bhattacharyya et al., 2013). Moreover, miR-155 targets the regulatory associated protein of mTOR complex 1 (*RPTOR*) and activates TGF-β signaling, and upregulates connective tissue growth factor (CTGF) in CF lung epithelial cells, thereby promoting fibrosis. (Tsuchiya et al., 2016). RPTOR is implicated in the modulation of the mammalian target of rapamycin complex 1 (mTORC1) activity that controls cell growth and survival whereas CTGF is a fibrotic factor that stimulates amplified fibrogenesis and airway remodeling. Furthermore, miR-16 rescues the F508del-CFTR trafficking defects probably through downregulation of heat shock protein 90 (HSP90) (Kumar et al., 2015).

**TABLE 4:** List of ncRNAs and their targets (other than CFTR) and functions in cystic fibrosis.

ncRNA	Source	Expression	Target/regulator	Function	Reference
miR-126	Bronchial brushing, 16HBE14o-, CFBE41o-, HEK293	down	TOM1	regulates NF-κB regulated IL-8 secretion	Oglesby et al. (2010)
miR-155	IB3-1, IB3-1/S9	up	SHIP1	upregulates IL-8 *via* regulation of PI3K/Akt signaling	Bhattacharyya et al. (2011)
miR-146a	16HBE14o- cells	down	MUC5AC	negative feedback role in the control of MUC5AC production	Zhong et al. (2011)
miR-145	Nasal epithelium cells, HEK293	up	SMAD3	downregulates SMAD3	Megiorni et al. (2013)
miR-155	IB3-1, IB3-1/S9	up	TTP, KSRP	TTP and KSRP regulate miR-155 biogenesis	Bhattacharyya et al. (2013)
miR-31	Differentiated primary cell cultures	down	IRF1	regulates a deteriorator of antimicrobial proteins, cathepsin S	Weldon et al. (2014)
miR-93	IB3-1, CuFi-1, NuLi-1	down	IL-8	regulates IL-8 *via* direct interaction	Fabbri et al. (2014)
miR-17	Bronchial brushing, 16HBE14o-, CFBE41o-, HEK293	down	IL-8	regulates IL-8	Oglesby et al. (2015)
miR-16	IB3-1, IB3-1/S9, CFPAC-1	basal comparable levels	HSP90	regulates F508del-CFTR trafficking defects	Kumar et al. (2015)
miR-199a-5p	Human and murine macrophages from lungs	up	CAV1	AKT/miR-199a-5p/CAV1 pathway as a regulator of innate immunity	Zhang et al. (2015)
miR-155	IB-3, IB3-1/S9	up	RPTOR	upregulates CTGF and regulates CF lungs fibrosis	Tsuchiya et al. (2016)
miR-1343	A549, 16HBE14o-, Caco-2	down	TGF-β	increases levels of activated TGF-β, pSMAD2 and pSMAD3	Stolzenburg et al. (2016)
miR-145	Primary cells from CF and non-CF patients	up	TGF-β	mediates TGF-β inhibition of CFTR synthesis and function	Lutful Kabir et al. (2018)
miR-199a-3p	CFBE41o-	down	IKKβ	increases IKKβ, NF-κB activity, and IL-8	Bardin et al. (2018)

In endobronchial brushings from CF patients, reduced expression of miR-126 upregulates the Target of Myb1 (TOM1) and regulates NF-κB-mediated IL-8 secretion (Oglesby et al., 2010). TOM1 belongs to a family of proteins containing an N-terminal VHS (Vps27p/Hrs/STAM) domain, and it has been demonstrated that TOM1 negatively regulates IL-1β- and TNF-α–induced signaling pathways while its upregulation leads to suppression of NF-κB (Yamakami and Yokosawa, 2004). Moreover, TOM1 through the interaction with Toll-interacting protein (Tollip) regulates intracellular trafficking (Yamakami et al., 2003; Katoh et al., 2004). These findings suggest miR-126, which directly targets TOM1, represents a crucial role in the regulation of innate immune responses and endosomal trafficking of ubiquitinated proteins in the CF lung. miR-146a negatively regulates Mucin 5AC (MUC5AC) expression, which is one of the foremost constituents of airway mucus, probably through the c-Jun N-terminal kinase (JNK) and NF-κB signaling in the neutrophil elastase (NE)-induced 16HBE14o-cells (Zhong et al., 2011). These results indicate the manipulation in miR-146a expression could regulate the excessive synthesis of mucus and thereby, CF pathogenesis. A recent study demonstrated that the inhibition of miR-146a induces increased expression of IL-6 in lipopolysaccharide (LPS)-stimulated CF macrophages (Luly et al., 2019). This study indicates that miR-146a dysregulation leads to dysfunctional CF macrophages, which results in impaired host defense and overproduction of inflammatory responses, and contributes to the progression and severity of CF. Several miRNAs including miR-509-3p, miR-494, and miR-126 regulate NF-kB, which in turn regulate CFTR expression and function (McKiernan et al., 2013; Ramachandran et al., 2013). Mir-31 downregulation increases cathepsin S, an inhibitor of antimicrobial proteins, through targeting the transcription factor *IRF-1* in CF pulmonary epithelial cells (Weldon et al., 2014). This results in the excessive accumulation of cathepsin S, which leads to the protease burden of the CF lung, and thereby the miR-31/IRF-1/CTSS pathway contributes to pulmonary inflammation in the CF airways (Weldon et al., 2014). The exogenous overexpression of miR-17 and miR-1343 downregulates IL-8 and TGF-β, respectively, in CF airway epithelial cells (Oglesby et al., 2015; Stolzenburg et al., 2016). MiR-199a-3p negatively regulates the NF-kB signaling pathway and IL-8 *via* its target inhibitor of nuclear factor kappa-B kinase subunit beta (*IKKβ*), which is implicated in the NF-κB pathway, and downregulation of miR-199a-3p contributes to pulmonary inflammation in the CF airways (Bardin et al., 2018).

Besides miRNAs, lncRNAs also regulate CF lung disease. For example, a number of lncRNAs including TLR8-AS1, HOTAIR, XIST, and MALAT are differentially expressed in bronchial brushings of CF patients (McKiernan et al., 2014). *Pseudomonas aeruginosa* infected CF bronchial epithelial cells exhibit dysregulation of several lncRNAs including MEG9 (maternally expressed 9) and BLACAT1 (bladder cancer-associated transcript 1) (Balloy et al., 2017). However, more investigations are required to understand the role and molecular mechanism of these lncRNAs in CF. Further, a recent study has illustrated the differential expression of lncRNAs in CF lung airway and parenchyma tissues, that affect multiple signaling pathways and cell membrane functions (Kumar et al., 2019). Interestingly, the suppression of lncRNA BGas rescues CFTR expression and function through the interaction with HMGB DNA-distorting proteins, members of the high mobility group (HMG) superfamily that lead to modifications of local chromatin and DNA structure of intron 11 of the CFTR gene (Saayman et al., 2016). Collectively, these observations underscore the promising associations of certain ncRNAs, both miRNAs and lncRNAs, in the direct or indirect regulation of CFTR expression and function, as well other aspects of CF disease phenotypes such as inflammation, airway obstruction, or infection as summarized in Tables 3, 4. The comprehensive knowledge of their roles and mechanisms in the pathogenesis and regulation of CF disease may represent a novel therapeutic approach for cystic fibrosis.

### Idiopathic Pulmonary Fibrosis (IPF)

IPF is a lethal progressive fibrotic disease of the lung interstitium, and is mainly characterized by persistent epithelial injury, scar tissue accumulation, increased fibroblast proliferation, amplified production of extracellular matrix (ECM), and excessive inflammation (Martinez et al., 2017; Mora et al., 2017). However, the exact etiology and pathogenesis of this disease is still not very well-defined.

In recent decades, the association between the pathogenesis of IPF and ncRNAs is recognized by an increasing number of studies. For example, miR-199a-5p is upregulated in lungs and lung myofibroblasts from IPF patients and bleomycin (BLM)-induced mouse models and activates lung fibroblast and fibrosis through targeting caveolin-1 (*CAV-1*), a major mediator of pulmonary fibrosis, and modulation of TGF-β signaling, which is involved in activation of fibroblasts proliferation and induction of EMT in alveolar epithelial cells (Lino Cardenas et al., 2013). MiR-21 is upregulated in peripheral blood from IPF patients, and inhibition of miR-21 in rat models upregulates its target a disintegrin-like and metalloproteinase with thrombospondin type 1 motif (*ADAMTS-1*), which downregulates pulmonary collagen type 1 (Col1) and collagen type 3 (Col3) contents and reduces the progression of IPF (Liu et al., 2016). The increased expression of miR-142-5p and reduced expression of miR-130a-3p are observed in macrophages from IPF patients and BLM-induced mouse models (Su et al., 2015). Thus, inhibition of miR-142-5p and overexpression of miR-130a-3p suppress lung fibrosis through stimulation of the STAT6 pathway by targeting peroxisome proliferator-activated receptor γ (*PPARγ*, a STAT6 coordinator) and suppressor of cytokine signaling 1 (*SOCS1*, a STAT6 inhibitor), respectively, which facilitates macrophage activation and contribute to extensive tissue fibrosis.

MiR-26a is downregulated in A549 cells and BLM-induced mouse models, and its overexpression diminishes epithelial-mesenchymal transition (EMT) through targeting high mobility group AT-hook 2 (*HMGA2*), a main positive regulatory factor in EMT (Liang et al., 2014a). The downregulation of miR-326 is reported in the lungs of IPF patients and BLM-induced mouse models (Das et al., 2014). Consistently, the overexpression of miR-326 suppresses TGF-β1 expression and diminishes the fibrotic response by downregulation of profibrotic genes (Ets1, Smad3, and matrix metalloproteinase 9 (MM9) and upregulation of antifibrotic genes (Smad7) (Das et al., 2014). The downregulation of miR-486-5p is found in lung tissues of IPF patients, and its overexpression in mouse models reduces lung fibrosis through targeting *SMAD2*, a crucial mediator of pulmonary fibrosis and implicated in TGF-β1 signaling (Ji et al., 2015). The downregulation of miR-323a-3p is found in lung epithelium from IPF patients, and its overexpression in IPF mouse models reduces fibroproliferation *via* directly targeting its targets *TGFA* and *SMAD*2 and modulation of various profibrotic signaling such as TGF-α, TGF-β, and apoptosis (Ge et al., 2016). In addition, miR-323a-3p downregulates CASP3, which prevents programmed cell death. MiR-221 is downregulated in tissues from human IPF, and in adenocarcinoma A549 and human bronchial epithelium (HBE) cell lines (Wang et al., 2016). Consistently, overexpression of miR-221 in these cell lines suppresses HMGA2 as well as phosphorylated-Smad3, which modulate TGF-β1 signaling, and leads to attenuation of EMT and lung fibrosis. The reduced expression of miR-29c is observed in alveolar epithelial cells from IPF patients, and overexpression of miR-29c in mice model reduces apoptosis, increases epithelial renewal, and thereby reduces lung fibrosis through targeting forkhead box O3a (*Foxo3a*), which is a transcription factor and play a crucial role in the induction of apoptosis (Xie et al., 2017). MiR-30a is downregulated in IPF patients, and further *in vitro* analyses indicate that overexpression of miR-30a directly targets ten-eleven translocation 1 (*TET1*) that modulates dynamin-related protein1 (Drp-1) promoter hydroxymethylation and thereby, show antifibrotic effect and defensive role against pulmonary damage (Zhang S. et al., 2017). The downregulation of miR-18a-5p is reported in pleural mesothelial cells (PMCs) and BLM-induced mouse models, and overexpression of miR-18a-5p downregulates its target *TGF-βRII* and reduces EMT of PMCs and sub-pleural pulmonary fibrosis (Zhang Q. et al., 2017). MiR-155 is downregulated in lung fibroblasts from IPF patients, and lung macrophages and fibroblasts from BLM-induced mouse models (Kurowska-Stolarska et al., 2017). In the same study, it is shown that overexpression of miR-155 decreases the exacerbated fibrotic response through downregulating its target liver X receptor α (*LXRα*), an oxysterol-activated transcription factor, and thereby, decreased production of collagen and TGF-β (Kurowska-Stolarska et al., 2017). The downregulation of miR-30a-5p is shown in exosomes from bronchoalveolar lavage fluid (BALF) of IPF elderly patients and A549 cells, its overexpression downregulates α-smooth muscle actin, and fibronectin expression by targeting TGF-β activated kinase 1/MAP3K7 binding protein 3 (*TAB3*), which is implicated in various cellular processes such as immune and inflammatory responses, altered fibrosis, and tissue repair and remodeling involved in IPF pathogenesis (Liu et al., 2018a). MiR-708-3p is downregulated in plasma and tissues from IPF patients, and overexpression of miR-708-3p attenuates lung fibrogenesis through directly modulating its target a disintegrin and metalloproteinase 17 (*ADAM17*), which regulates immune responses, fibrosis, and tissue regeneration, and by GATA/STAT3 signal pathway that is implicated in fibroblast-myofibroblast differentiation (Liu et al., 2018b). A recent report shows that miR-186 is downregulated in lung tissues of IPF patients, and delivery of miR-186 by human bone marrow mesenchymal stem cell-derived extracellular vesicles (BMSC-EVs) reduces fibroblast activation by downregulating its target SRY-related HMG box transcription factor 4 (*SOX4*) and thereby Dickkopf-1 (DKK1) (Zhou et al., 2021). SOX4 acts as a transcription factor and is associated with lung development and cell survival, whereas DKK1 is an inhibitor of the Wnt signaling pathway that have a significant role in lung development and differentiation and IPF pathogenesis and progression (Menezes et al., 2012; Guan and Zhou, 2017; Pan et al., 2017).

Several studies have also demonstrated the involvement of lncRNAs in the pathogenesis of IPF. For example, elevated levels of the lncRNA H19 in BLM-induced mouse models upregulates COL1A1 and Acta2, prominent factors linked with IPF pathogenesis, through direct targeting of miR-29b, and consequently knockdown of H19 attenuates fibrogenesis (Tang Y. et al., 2016). The telomeric repeat-containing RNA (TERRA) is upregulated in the blood of IPF patients, and further *in vivo* and *in vitro* studies demonstrate its role in fibrogenesis by the regulation of telomeric and mitochondrial functions (Gao et al., 2017). Dysfunctional telomerase activity and mitochondria under oxidative stress elicit apoptosis of epithelial cells and other processes linked with IPF progression. The increased expression of lncRNA PCF in the lungs of IPF patients induces pulmonary fibrosis by directly targeting miR-344a-5p and regulating map3k11 that elicit the proliferation of activated epithelial cells (Liu et al., 2017). Pulmonary fibrosis-regulatory lncRNA (PFRL) regulates the reciprocal repression of miR-26a and Smad2, which elicits the proliferation of activated epithelial cells, and contributes to the collagen deposition and progression of lung fibrosis (Jiang et al., 2018). In lungs and lung fibroblasts from mice, lncRNA pulmonary fibrosis-associated RNA (PFAR) functions as a competitive endogenous RNA (ceRNA) for miR-15a and in the regulation of yes-associated protein 1 (YAP1)-Twist expression, which is an important transcriptional effector in the Hippo pathway and implicated in the organ fibrosis process (Zhao et al., 2018; Sun et al., 2019). Upregulated lncRNA H19 in tissues from IPF patients downregulates miR-140 and modulates TGF-β/Smad3 signaling, and further *in vivo* and *in vitro* experiments show that the knockdown of H19 diminishes pulmonary fibrosis (Wang et al., 2019). The elevated expression of Zinc-finger E-box binding homeobox 1 antisense RNA 1 (ZEB1-AS1) and its positive correlation with the expression of ZEB1, which is a master regulator of EMT, is found in BLM-induced rats and TGF-β1-induced RLE-6TN cells (Qian et al., 2019). Subsequent experiments demonstrated that silencing of lncRNA ZEB1-AS1upregulates its target miR-141-3p and suppresses progression of EMT and fibrogenesis. Therefore, cumulatively these data clearly depict the regulatory functions, particularly the post-transcriptional regulation of ncRNA, miRNAs and lncRNAs, in onset, progression, and development of IPF. Table 5 summarizes the list of ncRNAs, their targets, and functions associated with IPF. Further, additional in-depth studies will lead to therapies for early diagnosis, control, and treatment of IPF.

**TABLE 5:** List of ncRNAs and their targets and functions in IPF.

ncRNA	Source	Expression	Target/regulator	Function	Reference
miR-199a-5p	lungs and lung myofibroblasts from IPF patients, BLM-induced mouse models	up	CAV1	mediates TGF-β induced lung fibroblast activation	Lino Cardenas et al. (2013)
miR-26a	A549 cells, BLM-induced mouse models	down	HMGA2	induces EMT	Liang et al. (2014a)
miR-26a	lungs from IPF patients, BLM-induced mouse models, MRC-5 cells	down	Smad4	reveals positive feedback loop between miR-26a and p-Smad3	Liang et al. (2014b)
miR-92a	lung fibroblasts from IPF patients, BLM-induced mouse models	down	WISP1	increases WISP1	Berschneider et al. (2014)
miR-326	lungs from IPF patients, BLM-induced mouse models, multiple human cell lines	down	TGF-β1	regulates TGF-β1 expression and other profibrotic genes (Ets1, Smad3, Smad7, and MM9)	Das et al. (2014)
miR-9-5p	lungs from IPF patients, BLM-induced mouse models	up	TGFBR2, NOX4	suppresses pro-fibrogenic transformation of fibroblasts and prevents organ fibrosis	Fierro-Fernandez et al. (2015)
miR-29c	lung tissue from IPF patients	down	type I collagen	dysregulates PP2A/HDAC4 axis and increases type I collagen expression	Khalil et al. (2015)
miR-130a-3p	macrophages from IPF patients, BLM-induced mouse models	down	PPARγ	regulates macrophage profibrogenic gene expression	Su et al. (2015)
miR-142-5p	macrophages from IPF patients, BLM-induced mouse models	up	SOCS1	regulates macrophage profibrogenic gene expression	Su et al. (2015)
miR-486-5p	lung tissues from IPF patients, silica-induced mouse models, BLM-induced mouse models	down	SMAD2	promotes lung fibrosis	Ji et al. (2015)
miR-21	Peripheral blood from IPF patients, BLM-induced rat models	up	ADAMTS-1	increases of pulmonary Col1 and Col3 contents and promotes progression of pulmonary fibrosis	Liu et al. (2016)
miR-26a	A549 and MLE-12 cells	down	Lin28B	induces EMT by inhibition of let-7d	Liang et al. (2016)
miR-27a-3p	lung fibroblasts from IPF patients	down	α-smooth muscle actin, Smad2, Smad4	functions *via* a negative-feedback mechanism in inhibiting lung fibrosis	Cui et al. (2016)
miR-29a	clinical specimens from IPF, MRC-5 cells	down	LOXL2, SERPINH1	causes overexpression of LOXL2 and SERPINH1	Kamikawaji et al. (2016)
miR-29c	lung fibroblasts, IPF lungs	down	Fas	causes resistance to Fas-mediated apoptosis	Matsushima and Ishiyama (2016)
miR-34a,b,c	type II AECs from IPF patients	up	E2F1, c-Myc, cyclin E2	regulates cellular senescence	Disayabutr et al. (2016)
miR-130b-3p	lungs from IPF patients	down	IGF-1	contributes to fibroblasts activation and dysregulated epithelial-mesenchymal crosstalk	Li et al. (2016)
miR-185, miR-186	lung from IPF patients, A549 and HCC827 cells	down	COL5A1	induces EMT and collagen V overexpression	Lei et al. (2016)
miR-221	tissues from human IPF, A549, HBE	down	HMGA2	induces EMT and pulmonary fibrosis	Wang et al. (2016)
miR-323a-3p	lung epithelium from IPF patients, BLM-induced mouse models	down	TGF-α, TGF-β, caspase-3	releases inhibition of various profibrotic pathways to promote fibroproliferation	Ge et al. (2016)
miR-338-5p	BLM-induced mouse models	down	SMO	induces EMT and contributes to fibrotic phenotype	Zhuang et al. (2016b)
miR-338-5p	BLM-induced mouse models	down	LPA1	promotes pulmonary fibrosis	Zhuang et al. (2016a)
miR-18a-5p	PMCs, BLM-induced mouse models	down	TGF-βRII	induces EMT of PMCs and sub-pleural pulmonary fibrosis	Zhang et al. (2017a)
miR-27b	lung fibroblasts from IPF patients, BLM-induced mouse models	down	TGF-βRI, SMAD2	stimulates fibroblast activation	Zeng et al. (2017)
miR-29c	AEC from IPF patients, BLM-induced mouse models	down	Foxo3a	increases apoptosis and reduces epithelial renewal	Xie et al. (2017)
miR-30a	IPF patients, MRC-5 cells	down	TET1	increases the TET1 and reduces Drp-1 promoter hydroxymethylation	Zhang et al. (2017b)
miR-34a	AEC from IPF patients, BLM-induced mouse models	up	p53	promotes lung epithelial injury and pulmonary fibrosis	Shetty et al. (2017)
miR-34a	lungs and lung myofibroblasts from IPF patients, BLM-induced mouse models	up	β-galactosidase, senescence markers	induces a senescent phenotype in lung fibroblasts	Cui et al. (2017)
miR-155	lung fibroblasts from IPF patients, lung macrophages and fibroblasts from BLM-induced mouse models	down	LXRα	increases exacerbated lung fibrosis, collagen deposition, TGF-β production	Kurowska-Stolarska et al. (2017)
miR-30a-5p	exosomes from BALF of IPF patients, A549 cells	down	TAB3	increases TAB3, α-smooth muscle actin and fibronectin expression	Liu et al. (2018a)
miR-708-3p	plasma and tissues from IPF patients	down	ADAM17	promotes fibrogenesis	Liu et al. (2018b)
miR-186	lung tissues from IPF patients	down	SOX4	miR-186 delivered by BMSC-EVs could suppress fibroblast activation	Zhou et al. (2021)
lncR-H19	BLM-induced mouse models	up	miR-29b	upregulates COL1A1 and Acta2 and promotes fibrogenesis	Tang et al. (2016a)
lncR-TERRA	blood from IPF patients, BLM-induced mouse models A549 and MLE-12 cells	up	genes/components associated with telomeres and mitochondria	regulates telomeric and mitochondrial functions	Gao et al. (2017)
lncR-PCF	lungs from IPF patients, BLM-induced rat models, RLE-6TN cells	up	miR-344a-5p	promotes pulmonary fibrogenesis	Liu et al. (2017)
lncR- PFRL	lungs and lung fibroblasts from BLM-induced mouse	up	miR-26a	contributes to progression of lung fibrosis by modulating the reciprocal repression between miR-26a and Smad2	Jiang et al. (2018)
lncR-H19	BLM-induced mouse models	up	miR-196a	promotes fibrogenesis	Lu et al. (2018)
lncR-PFAR	lungs and lung fibroblasts from BLM-induced mouse	up	miR-15a	modulates of YAP1-Twist expression	(Zhao et al., 2018; Sun et al., 2019)
lncR-H19	tissues from IPF patients, BLM-induced mouse models, HBE and A549 cells	up	miR-140	promotes pulmonary fibrosis *via* regulatory network of lncRNA H19-miR-140-TGF-β/Smad3 signaling	Wang et al. (2019)
lncR-ZEB1 -AS1	lungs from BLM-induced rat models, RLE-6TN cells	up	miR-141-3p	promotes EMT progress and fibrogenesis	Qian et al. (2019)

## Discussion

**FIGURE 1:**
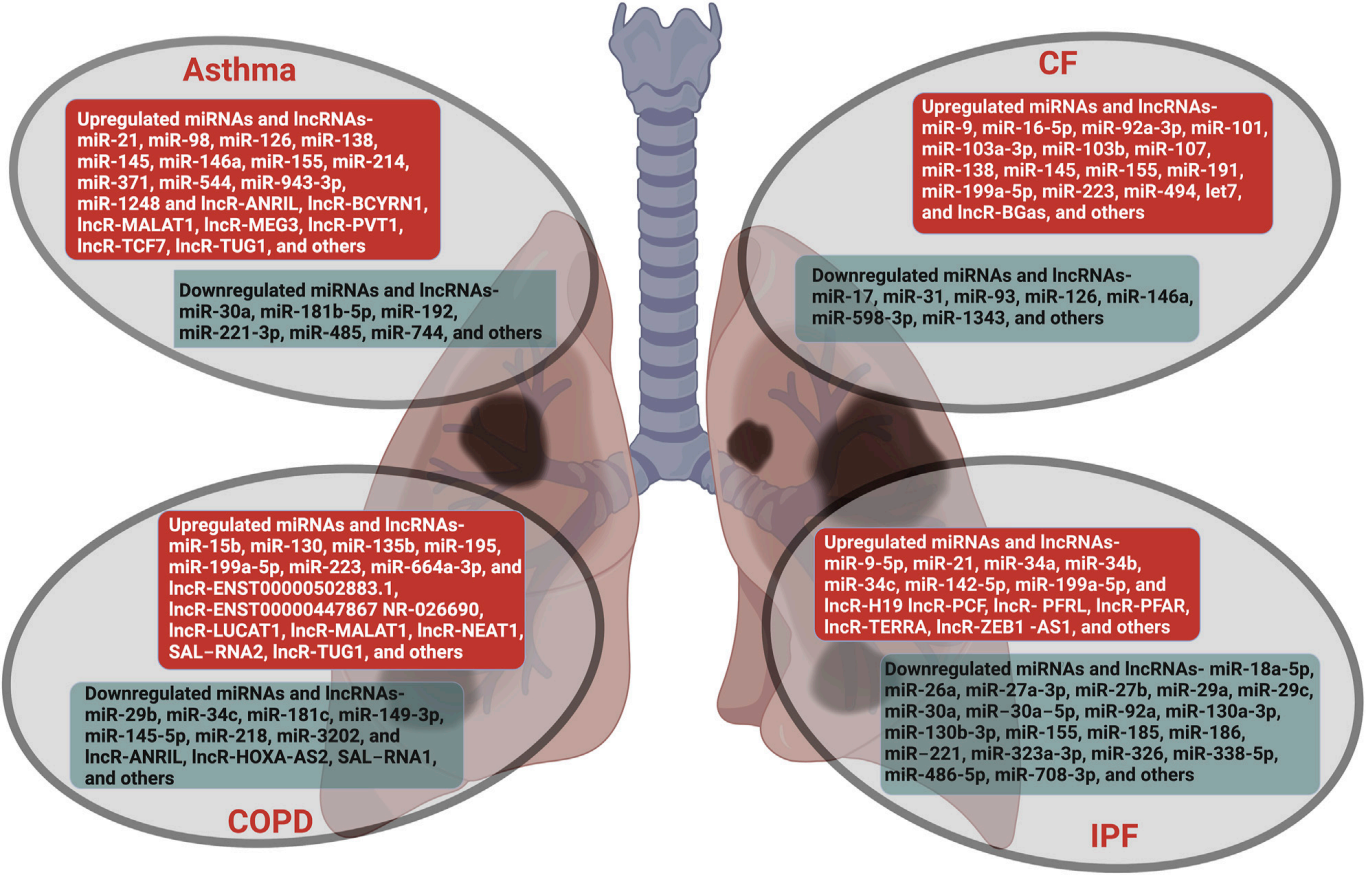
Lung diseases regulated by non-coding RNAs. The miRNAs and lncRNAs that are aberrantly expressed in lung diseases such as asthma, COPD, CF and IPF are listed. The ncRNAs that are upregulated are in red boxes and those that are downregulated are in green boxes.

Here, we summarize the emerging roles, post-transcriptional regulations, and mechanistic functions of ncRNAs, with emphasis on miRNAs and lncRNAs, in lung diseases that are a major public health concern. According to a recent report, only in the year 2017, lung diseases globally affected nearly 545 million people and caused 3.9 million deaths with an increase of 39.8 and 18.0%, respectively, since 1990 (Collaborators, 2020). Thus, lung diseases are a predominant cause of substantial morbidity and mortality worldwide and demand an exhaustive understanding of etiology and pathophysiology. Recent studies have established the association and regulatory function of ncRNAs in lung development and maintenance of lung homeostasis. The deregulation of ncRNAs causes pathophysiological alteration and contributes to the onset, progression, and development of various types of lung diseases such as asthma, COPD, CF, and IPF (Figure 1)

Alterations of miRNA and lncRNA expression level in the disease state compared to the normal state expedite a new paradigm for the diagnosis and appraisal of drug action. As presented in this review, disease-specific dysregulated miRNAs/lncRNAs are identified in various types of lung cells and tissues, which together with the higher stability of miRNAs mark them as clinical diagnostic biomarkers. However, a major challenge is the invasive procedures used for obtaining lung biopsies. Recent reports indicate detection of miRNAs/lncRNAs in body fluids such as blood, serum, plasma, BAL fluid, saliva, sputum, and urine, which have tremendous potential for relatively non-invasive diagnosis and prognosis of lung disease as well as appraisal of drug action. However, the utility of these biospecimen as a clinical diagnostic biomarker is yet to be examined and established with a larger patient cohort in various lung diseases.

Notably, ncRNA-based therapeutics have great potential in the treatment of lung diseases. Collectively, the studies summarized here show that enormous efforts have been made to deliver mimic or antisense oligonucleotide (ASO, including inhibitor, miRNA sponge, and target site blocker (TSB)) to overexpress or suppress specific genes that are downregulated or upregulated, respectively, in the diseased state and contribute to the pathogenesis and pathophysiology of lung diseases. However, in order to translate this treatment strategy from lab to clinical settings, some challenges including cell/tissue-specific delivery, stability and binding affinity, and off-target effects need to be addressed. Recent progress in generating modified derivatives of nucleic acid as potential drugs include numerous chemical strategies, such as the addition of 2′-O-methyl (2-O′-Me) or phosphorothioate-like groups, locked nucleic acids (LNA), miRNA sponges, nanoparticles, morpholinos, or peptide nucleic acids (PNA) as well as strategies for efficient delivery, such as viral vectors, polymers-, peptides-, and lipid-based delivery systems. Despite these efforts, there is still a need for more extensive studies to evaluate the effect of chemical modifications in *in vivo* systems and develop more consistent cell/tissue-specific delivery strategies.

Our current knowledge suggests that the identification of disease-specific miRNAs/lncRNAs and comprehensive knowledge of post-transcriptional regulation mechanisms will help understand their role and mode of functioning in the pathogenesis of lung diseases. Concurrently, the development of safe and cell/tissue-specific delivery systems will help to translate ncRNAs-based therapeutics from lab to clinical settings. Hence, we are optimistic that the continued elucidation of the function of ncRNAs encompasses the great potential to uncover diagnostic and prognostic tools and candidate therapeutic targets for lung diseases in the near future.

## Abbreviations

CF, Cystic Fibrosis; COPD, Chronic obstructive pulmonary disease; IPF, Idiopathic pulmonary fibrosis; LncRNA, Long non-coding RNA; miRNA, miR, MicroRNA.
